# How People Evaluate Anti-Corona Measures for Their Social Spheres: Attitude, Subjective Norm, and Perceived Behavioral Control

**DOI:** 10.3389/fpsyg.2020.567405

**Published:** 2020-11-12

**Authors:** Hendrik Godbersen, Laura Anna Hofmann, Susana Ruiz-Fernández

**Affiliations:** ^1^FOM Hochschule für Oekonomie & Management, Essen, Germany; ^2^Leibniz-Institut für Wissensmedien, Tübingen, Germany; ^3^LEAD Research Network, Eberhard Karls University of Tübingen, Tübingen, Germany

**Keywords:** COVID-19, COVID-19 and social spheres, anti-Corona measures, COVID-19 and theory of planned behavior, anti-Corona measures and theory of planned behavior, means-end theory of complex cognitive structures, COVID-19 and social groups, theory of planned behavior

## Abstract

Restrictions on outdoor activities, tips for hygiene, and tips for mental health are among the most common initiatives to counter the COVID-19 pandemic. These measures aim to protect people’s health and, at the same time, impact their social lives. So far, it is little known how people evaluate those anti-Corona measures with regard to their social spheres (close family, wider family and friends, colleagues, and society). Furthermore, it is plausible that the subjective evaluation of attitudinal objects and especially severe events, like the COVID-19 pandemic and the related counter-measures, is multidimensional. Against this background, we combine the social spheres with the elements of the Theory of Planned Behavior. On the methodological basis of the Means-End Theory of Complex Cognitive Structures, we determine the perceived relevance and quality of the attitude, subjective norm, perceived behavioral control, and social spheres regarding anti-Corona measures. Furthermore, the applied methodology allows the deduction of norm strategies to define the priority of securing or increasing the effectiveness of elements of anti-Corona measures. Based on the answers of 663 participants, we found that the protection from COVID-19 and its consequences (attitude) are more important to people than the practicability of anti-Corona measures in their social lives (perceived behavioral control), which, again, has a higher subjective relevance than the willingness to fulfill the expectations of others (subjective norm). Additionally, people distinguish between their close family (higher subjective relevance) and their other social spheres (lower subjective relevance). The people attribute the highest quality to the tips on hygiene, followed by the restrictions on outdoor activities and the tips for mental health. The protection and practicability of the anti-Corona measures have higher quality ratings than the willingness to fulfill the expectations of others. Based on the norm strategies, policymakers should secure the effectiveness of the current anti-Corona measures with a high priority by focusing on the protection and practicability with regard to close and wider family and friends. Increasing the effectiveness of the protection and practicability of anti-Corona measures in work and society also has a high priority. Focusing on the subjective norm should be of lower priority.

## Introduction

Our study examines the people’s evaluation of anti-Corona measures so that approaches for optimizing these measures can be derived. The necessary basis is laid in this section. After briefly characterizing the COVID-19 pandemic, we describe the main measures taken to counter the current crisis. To ensure a differentiated picture of how people evaluate the anti-Corona measures, we introduce the social spheres affected by the pandemic and the Theory of Planned Behavior, which will be combined to a hypothesized model.

### The COVID-19 Pandemic

The outbreak of coronavirus disease 2019 (COVID-19) caused by the novel severe acute respiratory syndrome coronavirus 2 (SARS-CoV-2) started in December 2019 in Wuhan, China, (ProMED-mail, 2019; ProMED-mail, 2020; WHO, 2020b) and rapidly spread in many countries all over the globe with a dramatically fast increase in new infections (Phan, 2020; Sohrabi et al., 2020; Velavan and Meyer, 2020). In March 2020, the World Health Organization WHO declared the coronavirus outbreak a pandemic (WHO, 2020c) with countries such as Spain, Italy, France, the United Kingdom and the United States being among the most-affected ones on a global level (Johns Hopkins University, 2020). At present (May 28, 2020), more than 5,550,000 cases of COVID-19 have been confirmed globally and more than 350,000 people have died from the disease (WHO, 2020a). The coronavirus is primarily transmitted from person to person via direct contact or respiratory droplets (Guerrero et al., 2020; Rothan and Byrareddy, 2020; Wang and Du, 2020). To date, a COVID-19-specific vaccine or therapeutic medication has not been developed, although many efforts in this direction have been made and are currently undertaken (Ahmed et al., 2020; Lurie et al., 2020; Le et al., 2020; Liu et al., 2020; Rothan and Byrareddy, 2020; Zhang et al., 2020b).

### Anti-Corona Measures

In the following two subsections, we will provide a general overview of anti-Corona measures and, then, focus on the German initiatives, as they are the research objects of our empirical analysis.

#### Overview of Anti-Corona Measures

As there is currently no vaccine or medication available for treating COVID-19, the current anti-Corona measures focus on slowing down the spread of SARS-CoV-2 and primarily contain reducing human social contacts and generating hygiene awareness (e.g., Balasa, 2020; Dalton et al., 2020; Jin et al., 2020; Kissler et al., 2020; Lewnard and Lo, 2020). In this context, numerous countries have introduced unprecedented measures labeled as “social distancing,” also called “spatial distancing,” which include, on the one hand, a general decrease of social contacts and, on the other hand, an increase of the space between people in order to minimize the risk of infection (Abel and McQueen, 2020; Kissler et al., 2020; Lewnard and Lo, 2020; Sen-Crowe et al., 2020). Some countries like Italy have enforced public distancing measures by imposing lockdowns (e.g., Sjödin et al., 2020); other countries like Sweden have taken less severe measures (e.g., Juranek and Zoutman, 2020). The objective of social or spatial distancing is to slow down the rate of infection and reduce the peak of incidence to a level the healthcare system is equipped to adequately respond to and save lives that would otherwise be left without treatment (Balasa, 2020; Sen-Crowe et al., 2020).

In an attempt to evaluate the initial impacts of anti-Corona measures, Bruinen de Bruin et al. (2020) were able to comprehensively categorize COVID-19 risk mitigation measures which are mobility restrictions, socioeconomic restrictions, physical distancing, hygiene measures, communication, and international support mechanisms. *Mobility restrictions* comprise limitations of public transport, air traffic, private cars, and outdoor activities (some countries only allowed walking outside with a dog or within a certain distance from home). *Socio-economic restrictions* target gatherings for educational, recreational, sportive, or work-related purposes (closing of shops, restaurants and bars, sports clubs, schools and universities, etc.). *Physical distancing* (also referred to as social or spatial distancing) means to maintain a proper distance of currently between 1.5 and 2 m to other people, prohibition of groups larger than 2–3 people, the closing of public spaces, etc. *Hygiene measures* aim to limit the spread of the virus and direct or indirect contamination of others (washing hands, sneezing, or coughing in elbow, avoiding touching face, contactless payments, wearing face masks, etc.). The cluster *communication* is the major drive for public understanding, trust, as well as acceptance and compliance with the measures introduced. Finally, *international support mechanisms* are important because the entire world is fighting the same threat and many countries have limited access to essential goods like medication or protection.

At present, it is hardly possible to clearly distinguish and evaluate the contribution of each cluster of measures to the overall decrease in new infections due to the lack of crucial information, namely, the case fatality rate, start and duration of infectiousness periods of COVID-19, and the existence of a large number of asymptomatic and undetected cases (Anderson et al., 2020). It is, however, believed that a combination of different mitigation measures, among others stopping mass gatherings, mobility restrictions, wearing appropriate face masks, screening programs, and the isolation of households, towns, or cities, could contribute to a faster decrease of new infections (e.g., Anderson et al., 2020; Balasa, 2020; Bruinen de Bruin et al., 2020). However, social or spatial distancing and hygiene measures seem to be at the core of such a mix of measures, promising the biggest effects (e.g., Bruinen de Bruin et al., 2020; Sen-Crowe et al., 2020).

The risk mitigation measures, mentioned above, can lead to enhanced levels of mental stress among individuals of the general population. Research suggests that social isolation, misinformation, and unpredictability and uncertainty about the seriousness of COVID-19 can contribute to stress and mental health concerns (Zandifar and Badrfam, 2020). Due to the permanent presence of inaccurate or exaggerated information, provided by media, and the perceived situation of mass threat, health anxiety and fear-related behaviors might arise and become excessive, possibly leading to maladaptive behaviors like hoarding or mistrust in authorities (Asmundson and Taylor, 2020). This can lead to risk exacerbation, for example evading medical treatment, and, as a consequence, accelerate the spread of COVID-19 (Espinola et al., 2016; Shultz et al., 2016).

The uncertainty of the current situation, the perceived mass threat, and feelings of isolation can lead to mental disorders, among others posttraumatic stress disorder, depression and anxiety disorders (e.g., Mak et al., 2009; Dar et al., 2017; Zhou et al., 2019; Xiang et al., 2020). Moreover, the isolation of people and the feeling of loneliness cannot only cause mental health issues but also negatively impact the physical health, for example cardiovascular problems, fragmented sleep, and diminished immunity (Leigh-Hunt et al., 2017; for a review, see Cacioppo and Cacioppo, 2014).

Thus, especially in countries with an elevated number of cases, quarantine measures, and isolated people, mental healthcare and psychological interventions should be incorporated in future disaster management plans all over the globe (Bao et al., 2020; Dong and Bouey, 2020; Duan and Zhu, 2020; Li et al., 2020; Xiang et al., 2020; Zhang et al., 2020a) in order to prevent long-term mental disorders, as was the case among SARS survivors (Mak et al., 2009). Against this backdrop, Mental Health Europe released tips for mental health in order to keep a sense of control and ease coronavirus anxiety (Mental Health Europe, 2020).

#### Anti-Corona Measures in Germany

We focus in our research on the social or spatial distancing and hygiene measures, which are at the core of virtually every country’s initiative to counter the COVID-19 pandemic, as well as on measures supporting the mental health of people. In our empirical analysis, we examine the subjective evaluation of these anti-Corona measures by the German population. Therefore, we describe the particular measures taken in Germany in more detail in the following paragraphs.

To mitigate the spread of COVID-19 in Germany, nationwide restrictions on public life were put into place on March 23, 2020. The government’s position was (Merkel, 2020): (1) Members of the public are required to reduce their contact with people other than the members of their own household to an absolute minimum. (2) In public, as far as possible, they must keep a distance of at least 1.5 m, preferably 2 m, from all those other than those mentioned in point number one. (3) Visiting public places is only permitted alone, with one other person who does not live in your household, or when accompanied by the members of your own household. (4) Travel to work or to provide emergency care, shopping for essentials, doctors’ appointments, attendance of meetings, necessary appointments and examinations, assistance for others, or sport and exercise individually out of doors as well as other necessary activities will, of course, still be possible. (5) Groups meeting for parties in public areas, homes, and private institutions are unacceptable in view of the serious situation in our country. Compliance with social distancing is to be monitored by the authorities responsible for public order and the police, and violations will be penalized. (6) Restaurants and cafés are to be closed. This does not include the delivery and collection of food that can be taken away and consumed at home. (7) Service providers in the personal care sector such as hairdressers, cosmetics studios, massage salons, tattoo parlors, and similar establishments are to be closed, because physical proximity is unavoidable in these professions, and this is not in line with the guidelines we have put in place for ourselves. Necessary medical treatments will still be permitted. (8) It is important that all enterprises, particularly those open to the public, adhere to the hygiene regulations and implement effective protective measures for staff and visitors. (9) These measures will apply for at least 2 weeks.

Those mitigation measures are complementing the following hygiene recommendations released by the Federal Centre for Health Education (BzgA, 2020): (1) Use a paper tissue or hold the crock of your arm in front of your mouth and nose when coughing or sneezing and dispose of the paper tissue immediately. (2) Keep your hand away from your face—do not touch your mouth, eyes, or nose with unwashed hands. (3) Stay away from individuals that have a cough, a cold, or fever—also because of the persistent wave of flue and cold infections. (4) Avoid touching (e.g., shaking hands or hugs) when greeting or saying goodbye to other people. (5) Wash your hands regularly and for a sufficient amount of time (at least 20 s) with soap and water—especially after blowing your nose, sneezing, or coughing.

Additionally, tips for mental health have been introduced by Mental Health Europe (2020), a European non-governmental network organization committed to the promotion of positive mental health across Europe (German version): (1) Seek accurate information from legitimate sources, for example WHO, European Commission, Robert Koch Institute, federal ministries, and public health offices. (2) Set limits around news on COVID-19. (3) Look after yourself including good hygiene, eating healthy, getting enough sleep, developing new daily routines for mental health, and doing things that you enjoy. (4) Reach out to others and support people around you (family, friends, people of need, people feeling lonely). This can benefit both the person receiving support as well as you as the helper. (5) Maintain a sense of hope and positive thinking, for example via focusing on positive news. (6) Acknowledge your feelings. Allow yourself to feel stressed, anxious, or depressed and express your feelings, for example in conversations or by writing them down. (7) Take time to talk with your children about the current situation to give them security. (8) Ask for professional support if necessary, for example at an advisory center or a self-help group.

### Social Spheres Affected by the Corona Pandemic

The description of anti-Corona measures, especially social distancing, makes it obvious that these measures do not only affect the individual but also the social lives of the people. Moreover, human life in general is characterized by events and encounters that develop over the course of time in connection to other individuals embedded within a social context (Elder et al., 2003). Human behavior—health related or not—can thus not be assessed without the individual’s specific social background and current social context. Germov (2014) suggests the coexistence of a biomedical model of health alongside a social model of health, highlighting that health and illness always occur within a specific social context.

Dahlgren and Whitehead (1991) describe the individual’s health influencing factors not only as inherent in age, sex, and genetic factors but also as embedded in an onion-like structured social environment consisting of the following determinants: (1) individual lifestyle (2) social and community influences, (3) living and working conditions, and (4) general socioeconomic, cultural, and environmental conditions. These determinants can be understood in the context of social groups, which can be specified and distinguished by different attributes, depending on the definition of a social group that is applied: shared experiences, status and roles of the group members, interactions, perception of being a group member etc. (e.g., Lewin, 1948; Bales, 1950; Sherif and Sherif, 1969; Tajfel, 1981). For example, Lickel’s et al. (2000) categorized groups in intimacy groups (e.g., families or friends), task-oriented groups (e.g., work groups or sports teams), social categories (e.g., Germans), and loose associations (e.g., people living in the same area).

Based on Lickel’s et al. (2000) categorization of social groups, we assume four social spheres to evaluate the social impact of the COVID-19 pandemic and the anti-Corona measures: close family, wider family and friends, colleagues at work, and society in general. The social spheres of close family and wider family and friends cover the intimacy groups. Colleagues at work represent the task group that, for most people, takes up the most of their time. The society in general can be roughly associated with a social category or a loose association. Thus, our categorization of social groups aims to cover the main social spheres affected by the COVID-19 pandemic and the referring counter measures.

### Theory of Planned Behavior as the Basic Structure for the Subjective Evaluation of Anti-Corona Measures

COVID-19 pandemic and the related counter measures represent a complex situation with potentially severe consequences for both the individuum and his or her social spheres, described in the previous section. The pandemic and its consequences cannot be controlled and eased without the cooperation of the population (Dayrit and Mendoza, 2020). Cooperation in this context can be translated into the people actively supporting the COVID-19 mitigation measures, introduced by governments and related organizations. Thus, the people’s positive evaluation of these measures with regard to their social spheres can be seen as pre-condition for their behavior and, therewith, the success of anti-Corona initiatives.

Social psychological theories, like the *Health Belief Model* (Becker, 1974; Rosenstock, 1974) and the *Theory of Planned Behavior* (Ajzen, 1985, 1988, 1991), provide a potentially fruitful framework to understand how people evaluate COVID-19 mitigation measures against the background of the current situation that can be perceived as complex and severe.

The Health Belief Model was applied in numerous health-related contexts, including the use of preventive screening and behaviors and compliance with medical regimes (for a review, see Sheeran and Abraham, 1996; Abraham and Sheeran, 2005), and is based on four main components: perceived susceptibility to a disease, perceived severity of a disease, perceived benefits of a specific preventive health action, and perceived costs of a specific preventive health action (Becker, 1974; Rosenstock, 1974). In the context of COVID-19, the Health Belief Model has already been applied to the topic of preventive communication of healthcare providers (Carico et al., 2020) as well as to the topic of its mental health and emotional impact (Mukhtar, 2020). For the present study, we decided against the Health Belief Model because it does not integrate social norms, which we believe are essential due to our focus on social spheres.

Instead, we apply the Theory of Planned Behavior which a number of studies suggest has more predictive power than the Health Belief Model (Bish et al., 2000; Lajunen and Räsänen, 2004; Şimşekoğlu and Lajunen, 2008).

The Theory of Planned Behavior is a social-cognitive model that stipulates the direct correlation between the individual’s behavioral intentions and his or her actual behavior (Ajzen, 1985, 1988, 1991). The Theory of Planned Behavior is an extension of the Theory of Reasoned Action (Fishbein and Ajzen, 1975; Ajzen and Fishbein, 1980), which is in turn based on the Fishbein model (Fishbein, 1963). Central idea of the Theory of Planned Behavior is that human behavior is determined by the following three constructs: *attitude*, *subjective norm*, and *perceived behavioral control*. *Attitude* is defined as an individual’s positive or negative evaluation of the consequences (benefits or drawbacks) of performing or not performing a specific behavior (Ajzen, 1988). *Subjective norm* refers to the degree of social pressure (opinion of significant others, e.g., peer pressure) an individual feels regarding the performance or non-performance of a specific behavior (Ajzen, 1988). *Perceived behavioral control* is an element extending the Theory of Reasoned Action (Ajzen, 1988) and describes an individual’s perception of personal capacities or constraints (factors like time, money, and chance) of performing a specific behavior.

The Theory of Planned Behavior provides a conceptual framework for determining the complexities of human behavior and has received empirical support in a wide range of applications in different domains (e.g., Manstead and Parker, 1995; Conner and Armitage, 1998; Armitage and Conner, 2001; Bamberg, 2003; Castanier et al., 2013). Studies have also demonstrated the predictive value of the Theory of Planned Behavior for understanding human decision-making processes that lead individuals to both pro-environmental (e.g., Boldero, 1995; Taylor and Todd, 1995; Cordano and Frieze, 2000; Holdsworth et al., 2019; Alzubaidi et al., 2020) and health conscious (e.g., Blue, 1995; Godin and Kok, 1996; Povey et al., 2000; O’Connor and Armitage, 2003; Lajunen and Räsänen, 2004; Şimşekoğlu and Lajunen, 2008) behaviors and decisions. The measures taken by governments to mitigate the spread of the Corona virus aim to protect both, the individuum and the society as a whole. As stated before, none of those measures would be effective without the collective contribution of every individuum. By complying with the measures applied (physical distancing, hygiene measures etc.), the individuals protect themselves while at the same time protecting others from infection with the Corona virus. In this context, the anti-Corona measures are comparable to pro-environmental as well as health-conscious behaviors.

The Theory of Planned Behavior (Ajzen, 1985, 1988, 1991) and its forerunner, the Theory of Reasoned Action (Fishbein and Ajzen, 1975; Ajzen and Fishbein, 1980), focus on the prediction of the behavioral intention by looking at the consequences of a specific behavior. The attitude, which is the central element of these theories, is modeled as the multiplication of the valance of a behavioral consequence with the probability that this consequence is an outcome of the behavior. This principal is not limited to the prediction of behavioral intentions but is also applied in other areas, e.g., motivation (Atkinson, 1964) or, as in Fishbein’s (1963) original theory, the evaluation of objects. It was also used to evaluate attitudinal objects and attitudinal structures within the Means-End Theory of Complex Cognitive Structures (Godbersen, 2016, 2019; Godbersen and Kaupp, 2019), which will be applied in our empirical research. In this context, the multiplicative model focuses on the subjective relevance of attributes of an attitudinal object and their perceived quality rather than the behavioral consequences.

In the previous section, we argued that the anti-Corona measures should be assessed with regard to four social spheres. Based on the content of this section, we propose that the subjective relevance and perceived quality of the attitude, subjective norm, and perceived behavioral control should be added to a model determining the people’s evaluation of anti-Corona measures.

### Hypothesized Model and Research Questions

Virtually all of the countries on the globe have taken measures to counter the spread of the Corona virus and ease its negative consequences on people’s health. The main measures to counter the Corona crisis in Germany are, among others, the restrictions on outdoor activities, tips for hygiene, and tips for mental health, as shown in Section “Anti-Corona Measures.” These three measures are examined in this paper.

In Section “Social Spheres Affected by the Corona Pandemic,” it was pointed out that the Corona virus and the referring counter-measures can impact different spheres of peoples’ social life: close family, wider family and friends, colleagues at work, and society in general. Furthermore, it was highlighted that one’s condition of health is not an individual phenomenon but should be understood in the context of the aforementioned social spheres. Therefore, we assume that the four social spheres, mentioned above, form the relevant context for people to evaluate anti-Corona measures.

People rarely evaluate attitudinal objects one-dimensionally or only based on one reason. Instead, the psychological evaluation of an object should be understood as a poly-causal process that contains multiple elements, even if people do not fully consciously go through this process. To account for this fact, the Theory of Planned Behavior (Ajzen, 1985, 1988, 1991) was introduced in Section “Theory of Planned Behavior As the Basic Structure for the Subjective Evaluation of Anti-Corona Measures.” Its three constructs, attitude, subjective norm, and perceived behavioral control, should form the main elements to evaluate the anti-Corona measures in our model.

**FIGURE 1:**
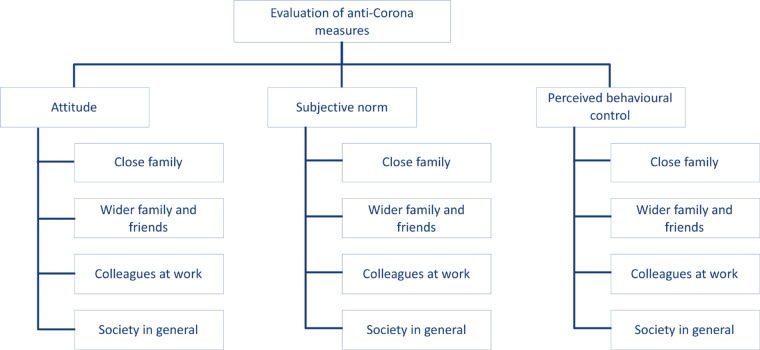
Model of people’s evaluation of anti-Corona measures (own representation).

Against this background, we propose a model (Figure 1), representing the people’s evaluation of anti-Corona measures, that consists of three levels. The overall evaluation of a measure to counter the Corona crisis is situated as a single construct on the top level. This can be the people’s evaluation of the restrictions on outdoor activities, tips for hygiene, and tips for mental health. The second level of the model consists of the attitude toward the respective measure, the subjective norm, and the perceived behavioral control in context with this measure. The social spheres—close family, wider family and friends, colleagues at work, and society in general—are situated on the third and most concrete level of the model. We assumed that these constructs are subordinated to the attitude, subjective norm, and perceived behavioral control when people evaluate anti-Corona measures. We also assume that the social spheres are of different relevance for the superordinated elements of our model.

In Section “Theory of Planned Behavior As the Basic Structure for the Subjective Evaluation of Anti-Corona Measures,” we did not only introduce the attitude, subjective norm, and perceived behavioral control as relevant constructs for a differentiated and comprehensive model of people’s evaluation of objects but also pointed out, in accordance with the Theory of Planned Behavior (Ajzen, 1985, 1988, 1991) and the more general expectancy value theories (e.g., Atkinson, 1964; Fishbein and Ajzen, 1975; Vroom, 1995), that the psychological overall evaluation of an object depends on the subjectively perceived relevance of its subordinate elements and their subjective assessment. Against this background and based on our hypothetical model, described above, we formulate the following research questions:

RQ1 (subjective relevance): Which relevance do the attitude, subjective norm, and perceived behavioral control as well as the social spheres have for the people’s evaluation of anti-Corona measures?

RQ1.1: Which relevance do the attitude, subjective norm, and perceived behavioral control have for the people’s evaluation of anti-Corona measures?

RQ1.2: Which relevance do the social spheres—close family, wider family and friends, colleagues at work, and society in general—have for the people’s evaluation of anti-Corona measures?

RQ2 (subjective quality): How well do people evaluate anti-Corona measures—restrictions on outdoor activities, tips for hygiene and tips for mental health—with regard to their attitude, subjective norm, and perceived behavioral control as well as their social spheres?

RQ2.1: How well do people evaluate anti-Corona measures—restrictions on outdoor activities, tips for hygiene, and tips for mental health—with regard to their attitude, subjective norm, and perceived behavioral control?

RQ2.2: How well do people evaluate anti-Corona measures—restrictions on outdoor activities, tips for hygiene, and tips for mental health—with regard to their social spheres?

RQ2.3: How well do people evaluate anti-Corona measures—restrictions on outdoor activities, tips for hygiene, and tips for mental health—overall?

RQ3 (optimization): What is the potential of and the need for increasing the effectiveness of anti-Corona measures from the people’s perspective, and with what priority should the current effectiveness of these measures be secured or increased with regard to attitude, subjective norm, and perceived behavioral control within the social spheres of people?

RQ3.1: From the people’s perspective, what is the potential of and the need for increasing the effectiveness of anti-Corona measures with regard to attitude, subjective norm, and perceived behavioral control within the social spheres of people?

RQ3.2: Based on the potential of and need for increasing the effectiveness of anti-Corona measures, with which priority should the effectiveness of anti-Corona measures be secured or increased with regard to attitude, subjective norm, and perceived behavioral control within the social spheres of people?

## Materials and Methods

The design and the measurement instruments of the empirical research are explained in this section.

### Research Design

A standardized online questionnaire was used to evaluate the subjective relevance and quality of the attitude, subjective norm, perceived behavioral control, and social spheres regarding anti-Corona measures. The data collection was realized from 25 March until 15 April. The data collection falls into the time when the German chancellor announced the restrictions on outdoor activities on 22 March and her following speech about easing these measures on 15 April. Participants of the study were students of FOM University of Applied Sciences in Germany. These students work in regular jobs and parallelly study business psychology. Eventually, the survey resulted in 663 completed questionnaires. The average age of the participants is 26.73 years with a standard deviation of 5.03. The youngest participant is 19 years, and the oldest 55 years of age. 25.34% are male and 74.66% female. 69.98% of the sample lives in a relationship while 30.02% are singles. 26.85% live in a single household, 47.66% live in a household with a second person, 13.27% live in a household of three persons, and 12.22% live in a household having four or more persons.

### Measurement With the Means–End Theory of Complex Cognitive Structures

At its core, the content of the questionnaire is based on the hypothesized model, presented in Section “Hypothesized Model and Research Questions.” To analyze this hierarchical system of cognitive representations on three levels, the Means–End Theory of Complex Cognitive Structures (Godbersen, 2016, 2019; Godbersen and Kaupp, 2019), which has its roots in the more general expectancy value theories (e.g., Atkinson, 1964; Fishbein and Ajzen, 1975; Vroom, 1995), was applied and is explained with regard to the evaluation of anti-Corona measures in the following subsections.

#### Subjective Relevance, Normed Values, and Total Normed Values

Measuring the subjective relevance, continuous rating scales, ranging from 0 (not important) to 100 (very important), were used. The participants were asked how important it is to them that anti-Corona measures lead to the protection from the disease and its consequences to measure the subjective relevance of the attitude. The subjective relevance regarding the subjective norm was operationalized through asking how important it is to the participants to fulfill the expectations of others during the Covid-19 epidemic. To measure the perceived behavioral control, the participants were asked how important the practicability of anti-Corona measures is for them. The subjective relevance of the social spheres—close family, wider family and friends, colleagues at work, and society in general—for the attitude, subjective norm, and perceived behavioral control were measured accordingly.

The analysis of the collected data starts with the calculation of the normed values. The normed values of the attitude, subjective norm, and perceived behavioral control are calculated through the following equation:

$$nV_{\text{i}}=\frac{V_{\text{i}}}{\sum_{i=1}^{n}V_{\text{i}}}$$

*nV*_*i*_, normed value of element *i* on the middle level of the model (attitude, subjective norm, and perceived behavioral control) for the evaluation of the overall anti-Corona measure.

*V*_*i*_, empirically determined subjective relevance of element *i* on the middle level of the model (attitude, subjective norm, and perceived behavioral control) for the evaluation of the overall anti-Corona measure.

The sum of all of the normed values (*nV*_*i*_) equates to 1 or 100%. The normed values can be understood—similar to a regression coefficient—as the strength of the impact the attitude, subjective norm, and perceived behavioral control has on the evaluation of an anti-Corona measure. The normed values of the social spheres for the aforementioned constructs are calculated in the same way so that the influence of the social spheres on the elements on the next (second) level of the model can be determined.

To determine the influence of the elements on the lowest level of the model (close family, wider family and friends, colleagues at work, and society in general within the attitude, subjective norm, and perceived behavioral control) on the element on the highest level (overall evaluation of an anti-Corona measure), the total normed values are calculated by applying the following equation:

$$tnV_{\text{j}}=nV_{\text{i}}*nV_{\text{j}}$$

*tnV*_*j*_, total normed value of element *j* on the lowest level of the model (close family, wider family and friends, colleagues at work, and society in general within the attitude, subjective norm, and perceived behavioral control).

*nV*_*i*_, normed value of element *i* on the middle level of the model (attitude, subjective norm, and perceived behavioral control).

*nV*_*j*_, normed value of element j on the lowest level of the model (close family, wider family and friends, colleagues at work, and society in general within the attitude, subjective norm, and perceived behavioral control).

As with the normed values (*nV*_*i*_) underneath an element of the next-higher level, the sum of the total normed values (*tnV*_*j*_) of all of the elements on the lowest level of the model equate to 1 or 100%. Therefore, the total normed value can be interpreted as the relative influence of an element on the lowest level of the model on the element on the highest level.

#### Subjective and Calculated Quality

The subjective quality of the anti-Corona measures was operationalized on the lowest level of the model, presented in Figure 1. The subjective quality regarding the restrictions on outdoor activities, the tips for hygiene, and the tips for mental health were evaluated in three different sections of the questionnaire. In each section, it was asked how good the respective anti-Corona measure is to protect the close family, wider family and friends, colleagues at work, and society in general (attitude); how well these groups evaluate the anti-Corona measure; and how practical the anti-Corona measure is for the participant of the questionnaire in these four social spheres. As a measurement tool, a continuous rating scale from 0 (not good) to 100 (very good) was used.

The quality of the attitude toward anti-Corona measures is calculated by summing up the empirically measured qualities of the subordinated social spheres (protection of the close family, wider family and friends, colleagues at work, and society in general) weighed with their respective normed values. This calculation is conducted accordingly for the qualities of the subjective norm and perceived behavioral control. The following equation represents the described procedure:

$$cQ_{\text{i}}=\sum_{j=1}^{n}\frac{V_{\text{j}}}{\sum_{j=1}^{n}V_{\text{j}}}*eQ_{\text{j}}$$

*cQ*_*i*_, calculated quality of element *i* on the middle level of the model (attitude, subjective norm, and perceived behavioral control).

*eQ*_*j*_, empirical quality of element *j* on the lowest level of the model (close family, wider family and friends, colleagues at work, and society in general within the attitude, subjective norm, and perceived behavioral control).

*V*_*j*_, perceived value of element *j* (empirically measured) on the lowest level of the model (close family, wider family and friends, colleagues at work, and society in general within the attitude, subjective norm, and perceived behavioral control).

The overall quality of an anti-Corona measure is calculated in the same way, using the calculated quality (*cQ*_*i*_) of the attitude, subjective norm, and perceived behavioral control and their normed values (*nV*_*i*_). Furthermore, the overall quality of each anti-Corona measure was empirically measured by asking the participants how good they evaluate the respective measure on a continuous rating scale from 0 (not good) to 100 (very good).

#### Norm Strategies

**FIGURE 2:**
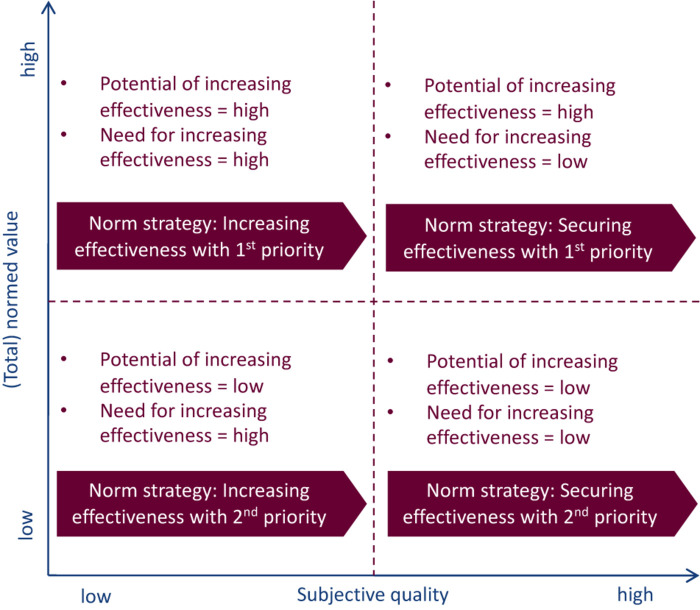
Norm strategies of the Means–End Theory of Complex Cognitive Structures depending on quality and (total) normed values (Godbersen, 2019).

Based on the afore-described analysis, the potential of and need for increasing the effectiveness of anti-Corona measures with regard to the attitude, subjective norm, and perceived behavioral control within the social spheres (close family, wider family and friends, colleagues at work, and society in general) and respective norm strategies can be “automatically” derived. The normed values and total normed values represent the relative influence that an element has on the overall evaluation of an anti-Corona measure. Therefore, the normed values and total normed values can be understood as being equivalent to the potential of increasing the effectiveness of anti-Corona measures. The subjective quality—empirical and calculated—of an element of the model corresponds with the need for increasing the effectiveness of anti-Corona measures regarding this specific element. The two described dimensions can be combined in a matrix, and norm strategies can be deduced, as shown in Figure 2. The four quadrants of the matrix are separated by the arithmetic mean of the normed values or total normed values and the arithmetic mean of the subjective quality.

#### Further Variables

Apart from the analysis with the Means–End Theory of Complex Cognitive Structures, the subjectively perceived level of information and the relevance of information sources during the Corona crisis as well as the perceived threat posed by the Covid-19 pandemic were measured. It was asked after the subjective level of information about the Covid-19 pandemic on a continuous rating scale from 0 (not good) to 100 (very good). The relevance of the close family, wider family and friends and colleagues at work, and relevance of classic media (television, newspapers, radio etc.) and new media (internet, social media etc.) as information sources for acquiring knowledge about the Covid-19 pandemic was measured on a continuous rating scale from 0 (not important) to 100 (very important). Furthermore, the perceived quality of information from the government and from researchers or research institutes was evaluated by using a continuous rating scale from 0 (not good) to 100 (very good). The perceived threat of the Covid-19 pandemic for the close family, wider family and friends, colleagues at work, and society in general was measured on a continuous rating scale from 0 (not threatening) to 100 (very threatening).

## Results

The presentation of the results is structured by four subsections. Firstly, the results for the subjective relevance and the (total) normed values of the elements of our model are described. Secondly, the subjective calculated qualities of the three examined anti-Corona measures and the subordinated elements are presented. Then, the two aforementioned categories of values are combined to “automatically” deduce norm strategies. Finally, the results for the additionally examined variables are described. All data were analyzed using R (R Development Core Team, 2017).

### Subjective Relevance and (Total) Normed Values of the Attitude, Subjective Norm and Perceived Behavioral Control, and Social Spheres

One of the research objectives of this paper is to determine the relevance people attribute to the attitude, subjective norm, and perceived behavioral control as well as to the social spheres in the context of anti-Corona measures (research question RQ1). To this end, the empirical values, normed values, and total normed values, which are represented in Table 1, are analyzed.

**TABLE 1:** Empirical, normed and total normed values (*n* = 663) (own representation).

Construct	Category	Empirical value		Normed value		Total normed value	
		Mean	*SD*	Mean	*SD*	Mean	*SD*
Attitude		87.84	16.79	0.39	0.08	0.39	0.08
Subjective norm		55.03	28.67	0.23	0.11	0.23	0.11
Perceived behavioral control		83.57	19.37	0.37	0.08	0.37	0.08
Close family (attitude)	Attitude	92.03	15.12	0.27	0.05	0.11	0.04
Wider family and friends (attitude)		86.52	18.90	0.25	0.03	0.10	0.02
Colleagues at work (attitude)		81.61	22.87	0.23	0.05	0.09	0.03
Society in general (attitude)		85.85	18.07	0.25	0.04	0.10	0.02
Close family (subjective norm)	Subjective norm	65.57	29.13	0.30	0.11	0.07	0.04
Wider family and friends (subjective norm)		52.78	29.71	0.23	0.07	0.05	0.03
Colleagues at work (subjective norm)		52.92	29.65	0.23	0.09	0.05	0.03
Society in general (subjective norm)		52.18	29.40	0.23	0.10	0.05	0.03
Close family (perceived behavioral control)	Perceived behavioral control	86.85	18.80	0.29	0.08	0.11	0.04
Wider family and friends (perceived behavioral control)		74.33	25.51	0.24	0.06	0.09	0.03
Colleagues at work (perceived behavioral control)		74.31	25.89	0.23	0.06	0.09	0.03
Society in general (perceived behavioral control)		75.28	24.78	0.24	0.07	0.09	0.03

The attitude (the perceived protection from the coronavirus and its consequences) is slightly more important than the perceived behavioral control in the social lives of people (the practicability of anti-Corona measures in one’s social life). Of the least importance to people is the subjective norm (the drive or willingness to fulfill the expectations of others).

With regard to the social spheres, the close family is of highest relevance to the participants of our survey. The empirical, normed, and total normed values of the wider family and friends, the colleagues at work, and the society in general are on a lower level with similar arithmetic means. This pattern can be observed in all of the three categories—attitude, subjective norm, and perceived behavioral control. It should be noted, however, that the social spheres, subordinated to the attitude and the perceived behavioral control, have a higher impact on the overall evaluation of anti-Corona measures than those subordinated to the subjective norm. This is due to the fact that the attitude and the perceived behavioral control themselves are of higher subjective relevance to the participants of the survey than the subjective norm.

### Subjective Calculated Quality of the Attitude, Subjective Norm and Perceived Behavioral Control, and Social Spheres

The second main research objective of this study is to determine how well people evaluate anti-Corona measures—restrictions on outdoor activities, tips for hygiene, and tips for mental health—with regard to their attitude, subjective norm, and perceived behavioral control as well as their social spheres (research question RQ2).

**FIGURE 3:**
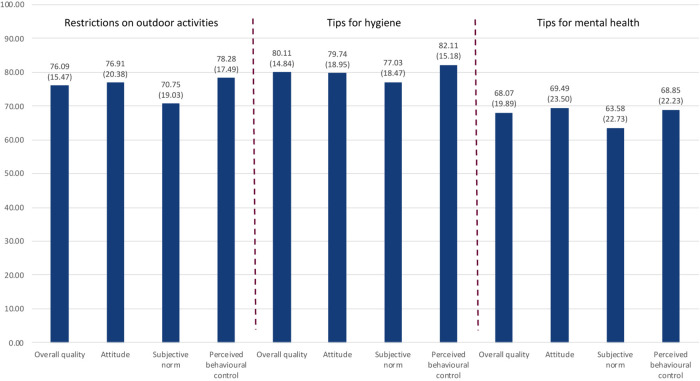
Calculated quality of the anti-Corona measures and their subordinated constructs—attitude, subjective norm, and perceived behavioral control (*n* = 663, arithmetic mean as number, standard deviation in brackets) (own representation).

The subjectively perceived qualities of the restriction on outdoor activities, tips for hygiene, and tips for mental health, calculated according to the Means–End Theory of Complex Cognitive Structures are represented by the first bars of each section in Figure 3. This figure also shows the calculated qualities of the attitude, subjective norm, and perceived behavioral control for each of the three anti-Corona measures.

Considering the range of the applied scale from 0 (not good) to 100 (very good), all of the three measures are evaluated rather positively. The tips for hygiene are evaluated best, followed by the restrictions on outdoor activities. The lowest quality is attributed to the tips for mental health.

To validate the calculated qualities of the anti-Corona measures and, with it, the overall model, the participants had to rate the three measures on a scale from 0 (not good) to 100 (very good). An arithmetic mean of 77.98 (*SD* = 21.25) resulted for the restrictions on outdoor activities, an arithmetic mean of 79.39 (*SD* = 19.34) for the tip for hygiene and for the tips for mental health an arithmetic mean of 68.34 (*SD* = 24.08). The differences between calculated and empirical values range between −1.89 and 0.72. Considering the scale from 0 to 100, this indicates a high validity of the measurements and calculations with the Means–End Theory of Complex Cognitive Structures. Furthermore, we confirmed the adequacy of our models by conducting partial least square path modeling, using the R package plspm (Sanchez, 2013), and calculating the variance inflation factors, using the R package faraway (Faraway, 2016); the results can be found in the Supplementary Materials.

For each anti-Corona measure, the qualities of the attitude, subjective norm, and perceived behavioral control show the same pattern. The attitude (the perceived protection from the Corona virus and its consequences) and the perceived behavioral control (the practicability of anti-Corona measures in one’s social life) are evaluated roughly on the same level and are better assessed than the subjective norm (the drive or willingness to fulfill the expectations of others).

The subjectively perceived qualities of the three anti-Corona measures with regard to the attitude, subjective norm, and perceived behavioral control within the social spheres are presented in Table 2.

**TABLE 2:** Empirical quality of the social spheres within the attitude, subjective norm, and perceived behavioral control for the anti-Corona measure restrictions on outdoor activities, tips for hygiene, and tips for mental health (*n* = 663) (own representation).

Construct	Category	Restrictions on outdoor activities		Tips for hygiene		Tips for mental health	
		Mean	*SD*	Mean	*SD*	Mean	*SD*
Attitude: close family	Attitude	79.61	22.62	80.58	22.08	72.54	25.08
Attitude: wider family and friends		78.62	22.21	81.24	20.45	70.39	25.09
Attitude: colleagues at work		71.74	27.36	77.95	22.95	67.46	26.49
Attitude: society in general		75.67	23.67	78.22	22.40	66.12	25.79
Subjective norm: close family	Subjective norm	77.93	21.72	81.48	19.84	67.60	25.24
Subjective norm: wider family and friends		70.34	23.31	77.96	20.44	64.39	24.91
Subjective norm: colleagues at work		67.24	25.61	75.86	22.87	61.72	25.31
Subjective norm: society in general		65.31	20.92	71.70	21.28	59.57	23.87
Perceived behavioral control: close family	Perceived behavioral control	82.24	23.75	82.82	22.55	75.16	25.01
Perceived behavioral control: wider family and friends		84.29	23.37	87.18	17.87	69.95	25.31
Perceived behavioral control: colleagues at work		70.84	31.40	80.60	23.61	65.23	27.23
Perceived behavioral control: society in general		73.18	24.13	76.50	22.85	61.36	27.14

The people’s evaluation of the protection from the Corona virus and its consequences (attitude) through the three measures is better for the social spheres of close family and wider family and friends than for colleagues at work and the society in general. Within the category of the subjective norm, the perceived qualities of the social spheres have the following descending order: close family, wider family and friends, colleagues at work, and the society in general. The practicability of the measures (perceived behavioral control) is rated higher for the close family and the wider family and friends than for the colleagues at work and the society in general.

### Norm Strategies for Optimizing Anti-Corona Measures

The third main research objective concerns the determination of the potential and need for optimizing elements of the anti-Corona measures and, based on that, deducing norm strategies for which elements the effectiveness should be secured or increased and with which priority (research question RQ3).

#### Norm Strategies for the Restriction on Outdoor Activities

**FIGURE 4:**
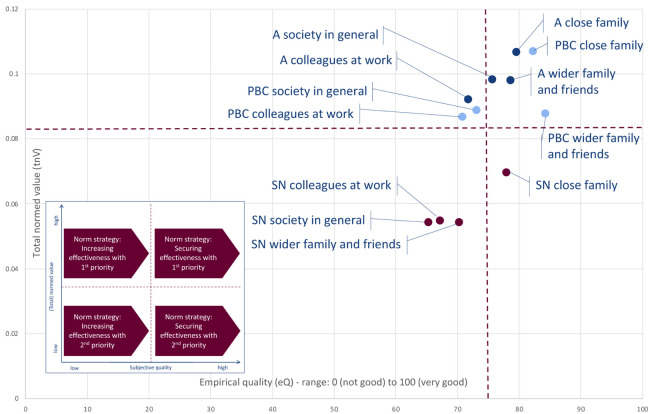
Empirical quality and total normed values of the attitude (A), subjective norm (SN), and perceived behavioral control (PBC) within the social spheres regarding the restrictions on outdoor activities (*n* = 663) (own representation).

The total normed values (tnV) and the subjective qualities (eQ) of the attitude, subjective norm, and perceived behavioral control for the four social spheres—close family, wider family and friends, colleagues at work, and society in general—regarding the restrictions on outdoor activities are presented in the form of a matrix in Figure 4.

According to the norm strategies that can be deduced from Figure 4, the effectiveness of the restriction on outdoor activities should be mainly secured with higher priority with regard to the following elements (Figure 4, top right quadrant):

– Attitude within the social sphere of the close family.
– Perceived behavioral control within the social sphere of the close family.
– Attitude within the social sphere of the wider family and friends.
– Perceived behavioral control within the social sphere of the wider family and friends.
– Attitude within the social sphere of the society in general.

The effectiveness of the restriction on outdoor activities should be mainly increased with higher priority with regard to the following elements (Figure 4, top left quadrant):

– Attitude within the social sphere of the colleagues at work.
– Perceived behavioral control within the social sphere of the colleagues at work.
– Perceived behavioral control within the social sphere of the society in general.

With a lower priority, the effectiveness of the social norm within close families should be mainly secured (Figure 4, bottom right quadrant).

The effectiveness of the restriction on outdoor activities should be mainly increased with a lower priority with regard to the following elements (Figure 4, bottom left quadrant):

– Subjective norm within social sphere of the wider family and friends.
– Subjective norm within social sphere of the colleagues at work.
– Subjective norm within social sphere of the society in general.

#### Norm Strategies for the Tips for Hygiene

**FIGURE 5:**
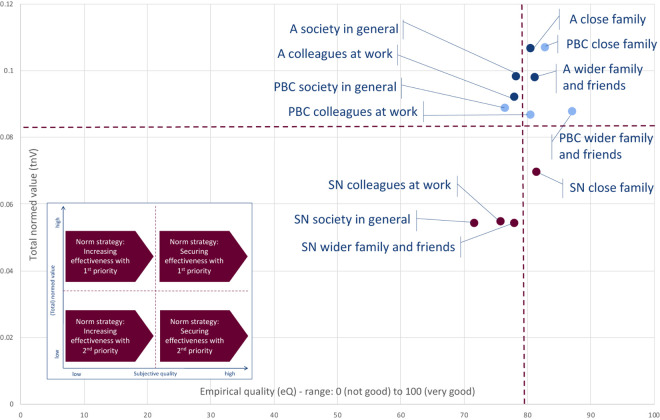
Empirical quality and total normed values of the attitude (A), subjective norm (SN), and perceived behavioral control (PBC) within the social spheres regarding the tips for hygiene (*n* = 663) (own representation).

The total normed values (tnV) and the subjective qualities (eQ) of the attitude, subjective norm, and perceived behavioral control for the four social spheres—close family, wider family and friends, colleagues at work, and society in general—regarding the tips for hygiene are presented in Figure 5.

According to the norm strategies that can be deduced from Figure 5, the effectiveness of the tips for hygiene should be mainly secured with higher priority with regard to the following elements (Figure 5, top right quadrant):

– Attitude within the social sphere of the close family.
– Perceived behavioral control within the social sphere of the close family.
– Attitude within the social sphere of the wider family and friends.
– Perceived behavioral control within the social sphere of the wider family and friends.
– Perceived behavioral control within the social sphere of the colleagues at work.

The effectiveness of the tips for hygiene should be mainly increased with higher priority with regard to the following elements (Figure 5, top left quadrant):

– Attitude within the social sphere of the colleagues at work.
– Attitude within the social sphere of the society in general.
– Perceived behavioral control within the social sphere of the society in general.

With a lower priority, the effectiveness of the social norm within close families should be mainly secured (Figure 5, bottom right quadrant).

The effectiveness of the tips for hygiene should be mainly increased with a lower priority with regard to the following elements (Figure 5, bottom left quadrant):

– Subjective norm within the social sphere of the wider family and friends.
– Subjective norm within the social sphere of the colleagues at work.
– Subjective norm within the social sphere of the society in general.

#### Norm Strategies for the Tips for Mental Health

**FIGURE 6:**
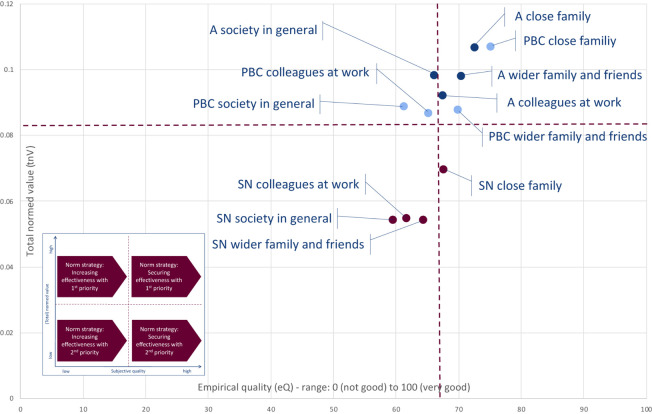
Empirical quality and total normed values of the attitude (A), subjective norm (SN), and perceived behavioral control (PBC) within the social spheres regarding the tips for mental health (*n* = 663) (own representation).

Figure 6 shows the total normed values (tnV) and the subjective qualities (eQ) of the attitude, subjective norm, and perceived behavioral control for the four social spheres—close family, wider family and friends, colleagues at work, and society in general—regarding the tips for mental health.

According to the norm strategies that can be deduced from Figure 6, the effectiveness of the tips for mental health should be mainly secured with higher priority with regard to the following elements (Figure 6, top right quadrant):

– Attitude within the social sphere of the close family.
– Perceived behavioral control within the social sphere of the close family.
– Attitude within the social sphere of the wider family and friends.
– Perceived behavioral control within the social sphere of the wider family and friends.
– Attitude within the social sphere of the colleagues at work.

The effectiveness of the tips for mental health activities should be mainly increased with higher priority with regard to the following elements (Figure 6, top left quadrant):

– Attitude within the social sphere of the society in general.
– Perceived behavioral control within the social sphere of the colleagues at work.
– Perceived behavioral control within the social sphere of the society in general.

With a lower priority, the effectiveness of the social norm within close families should be mainly secured (Figure 6, bottom right quadrant).

The effectiveness of the tips for mental health should be mainly increased with a lower priority with regard to the following elements (Figure 6, bottom left quadrant):

– Subjective norm within the social sphere of the wider family and friends.
– Subjective norm within the social sphere of the colleagues at work.
– Subjective norm within the social sphere of the society in general.

### Perceived Information Level, Information Sources and Perceived Threat

**FIGURE 7:**
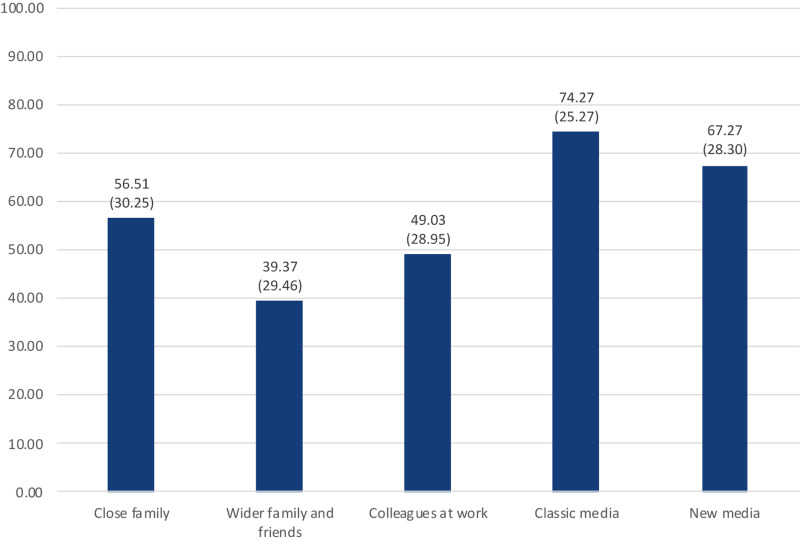
Relevance of information sources on a scale from 0 (not important) to 100 (very important) (*n* = 663, arithmetic mean as number, standard deviation in brackets) (own representation).

The perceived level of information about the Covid-19 pandemic, the perceived relevance of information sources, the perceived quality of governmental and research information, and the perceived threat of the Covid-19 pandemic were measured to gain additional insights in the people’s evaluation of the Corona crisis. Overall, people feel rather well informed about the Covid-19 pandemic which is indicated by an arithmetic mean of 69.16 (*SD* = 21.03) on a scale from 0 (not good) to 100 (very good). The subjectively perceived relevance of information sources is represented in Figure 7. The highest relevance—measured on a scale from 0 (not important) to 100 (very important)—has classic media followed, in descending order, by new media, close family, colleagues at work, and the wider family and friends. On a scale from 0 (not good) to 100 (very good), the information from the government is rated 64.30 (*SD* = 23.66) on average and the information from researchers and research institutes is rated 69.84 (*SD* = 24.49) on average.

**FIGURE 8:**
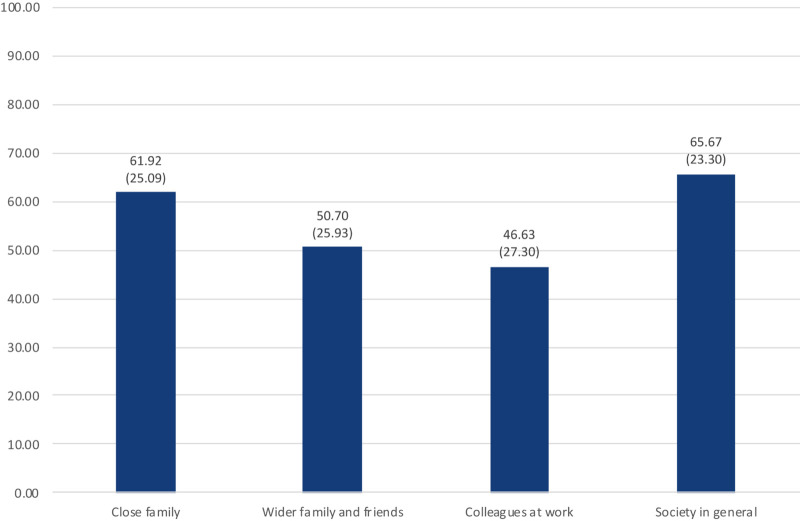
Perceived threat for the social spheres on a scale from 0 (not threatening) to 100 (very threatening) (*n* = 663, arithmetic mean as number, standard deviation in brackets) (own representation).

The perceived threat of the Covid-19 pandemic for the social spheres, which was measured on a scale from 0 (not threatening) to 100 (very threatening), is presented in Figure 8. The participants of the survey see the largest threat for the society in general, followed by the perceived threat to the close family. The threats to the wider family and friends, and the colleagues at work, are perceived on a lower level.

## Discussion

The objectives of our study, as presented in Section “Hypothesized Model and Research Questions,” are examining the people’s relevance [discussed in section “People’s Expectations on (Governmental) Initiatives and Measures”] and evaluation [discussed in section “People’s Evaluation on (Governmental) Initiatives and Measures”] of the main anti-Corona measures (restrictions on outdoor activities, tips for hygiene, and tips for mental health) as well as deducing approaches for optimizing these measures [discussed in section “Improving (Governmental) Initiatives and Measures”]. To gain differentiated insights in the three aforementioned areas we focus, following the Theory of Planned Behavior, on the protection from COVID-19 and its consequences (attitude), the practicability of the anti-Corona measures (perceived behavioral control) and the willingness to fulfill the expectations of others (subjective norm). Furthermore, we also integrate the social spheres of the close family, the wider family and friends, the colleagues at work, and the society in general in our study.

### People’s Expectations on (Governmental) Initiatives and Measures

The empirical and normed values revealed that the perceived protection from the Corona virus and its consequences (attitude) is slightly more important to the people than the practicability of the anti-Corona measures (perceived behavioral control), which in turn has a substantially higher subjective relevance than the willingness to fulfill the expectations of others (subjective norm).

Interestingly, other studies came to the result that the attitude and subjective norm are more important than the perceived behavioral control to predict health beneficial behavior. That is shown by the studies of Lajunen and Räsänen (2004) and Şimşekoğlu and Lajunen (2008) which examined the intention to use bicycle helmets and seat belts, behaviors that primarily have an impact on the individual health. We, on the other hand, study the people’s evaluation of measures that are aimed to stop the Corona virus from “socially” spreading. Thus, it is surprising that the subjective norm, as a social construct, is substantially less important to people in this context.

The order of the subjective relevance—protection from the coronavirus and its consequences (attitude) over the perceived practicability of the anti-Corona measures (perceived behavioral control) and substantially over the willingness to fulfill the expectations of others (subjective norm)—indicates that people judge initiatives in context with the COVID-19 pandemic by their effectiveness and efficiency rather than by social influence or even social pressure. This structure of subjective relevance can be understood as people’s expectations or preferences regarding (governmental) measures and initiatives that deeply impact people’s lives and even cut their fundamental civil rights. Therefore, policymakers and other relevant institutions should primarily focus on the utility for people (in this case protection from the COVID-19 pandemic and the practicability of anti-Corona measures in the people’s lives), when designing campaigns countering severe events like the COVID-19 pandemic.

Furthermore, the order of subjective relevance also indicates that people individually assess anti-Corona measures—at least they believe that they do so—and do not primarily form their opinion based on social interactions. This is backed by the relevance that people attribute to information sources during the COVID-19 pandemic: classic media is most important, followed by new media, which is, in turn, more important than social interactions with family members, friends, and colleagues (see section “Perceived Information Level, Information Sources and Perceived Threat”). Therefore, policymakers should comprehensively and factually communicate and explain the measures they are imposing on citizens. Our data suggests that this approach leads to convincing people of the necessity of strict and severe measures rather than communication campaigns incorporating social pressure, like “what would your grandmother say,” which is contrary to deductions of other researchers who see the most efficient way of changing health beneficial behavior in influencing the opinion of peers (Lajunen and Räsänen, 2004). This, however, might be a culturally sensitive aspect. We collected our data in Germany, a country with a rather individualistic culture; the results might differ in countries with a rather collectivistic culture and a stronger focus on social groups other than just the closest family (Triandis, 1995).

With regard to the social spheres, our data revealed that the close family is of higher subjective relevance to people than the wider family and friends, the colleagues at work, and the society in general, when it comes to evaluating anti-Corona measures. In the context of the COVID-19 pandemic, the close family is of highest relevance to the people even though they perceive a higher threat level for the society in general than for the close family and the other social groups (see section “Perceived Information Level, Information Sources and Perceived Threat”). This fact is, however, not surprising, as the close family normally is the group with the highest emotional closeness. What is, however, surprising is that the subjective relevance of the wider family and friends is perceived on a similar level as the subjective relevance of the colleagues at work and the society in general. One might expect that the emotional closeness and consequently the relevance of the former group are higher. An explanation might be found in the tendency of developed societies to emphasize more on individualistic values so that people predominantly focus on themselves and their small families (Triandis, 1995). Furthermore, cultural aspects might have an influence on these results, as mentioned above.

The highest subjective relevance of the close family implies that the people’s expectations on (governmental) initiatives that deeply impact their lives in situations like the COVID-19 pandemic are mainly focused on the protection of their close family and the practicability within this social sphere. Therefore, in a first step policymakers and related institutions need to design and communicate such initiatives with two main questions in mind: How do the small families benefit from the measures and how can small families integrate these measures in their daily lives with relative ease and without too many hurdles? In other words, the close family should be at the core of initiatives like the recent and current anti-Corona measures. It is, however, not sufficient to only focus on the small family. The values of the subjective relevance for the wider family and friends, colleagues at work, and society in general suggest that these social spheres are not as important as the close family but cannot be disregarded from the people’s perspective. Thus, the benefits for these social spheres and the referring practicability of measures need to be included as “secondary” aspects in the design and communication of severe (governmental) initiatives.

### People’s Evaluation on (Governmental) Initiatives and Measures

As described in Section “Subjective Calculated Quality of the Attitude, Subjective Norm and Perceived Behavioral Control, and Social Spheres,” the measures against the COVID-19 pandemic and its consequences, taken in Germany, are perceived rather well by the people. This indicates that people accept measures with large impacts on their lives, including the restriction of fundamental civil rights, in the face of a threat that is perceived as being dangerous. The tips for hygiene are evaluated best, followed by the restrictions on outdoor activities which also find a relatively high level of approval. The tips for hygiene are rated positively but with a gap to the measures mentioned before. The tips for hygiene and the restrictions on outdoor activities aim to protect people from infections with the coronavirus while the tips for mental health focus on easing rather “intangible” psychological consequences that might occur in the long run. This indicates that people focus more on the immediate threats of severe events than on the long-term consequences.

The protection from COVID-19 and its consequences (attitude), the practicability of the anti-Corona measures (perceived behavioral control), and the willingness to fulfill the expectations of others (subjective norm) show the same pattern across the three examined anti-Corona measures. The protection from COVID-19 and its consequences and the practicability of measures in people’s lives are on a similar quality level which is higher than the quality of the willingness to fulfill the expectations of others. This pattern roughly mirrors the pattern of the subjective relevance which can lead to two conclusions. On the one hand, it can indicate that the measures were designed and communicated according to the expectations of people. On the other hand, it can mean that the extensive media coverage, the statements of governmental officials, and the public discussion of the COVID-19 pandemic, focusing on the threats and spreading of the virus and, with it, the necessity and benefits of hygiene and social or spatial distancing measures, have influenced the people’s expectations. This explanation also corresponds with our finding that classic media is the most important source for the people to be informed about the COVID-19 pandemic (see section “Perceived Information Level, Information Sources and Perceived Threat”). Comparable results were found for the years after the terrorist attacks on September 09, 2001 when the media coverage and statements of the United States President and other United States officials positively correlated with the terrorism threat perceived by the American people (Nacos et al., 2007).

Across the three anti-Corona measures (restrictions on outdoor activities, tips for hygiene, and tips for mental health), the quality ratings for the close family and the wider family and friends, the two groups with a normally smaller size and closer emotional bonds, are higher than for the social groups of colleagues at work and the society in general. The characteristics of the former groups might lead people to believe that their individual behavior to counter the COVID-19 pandemic has a larger effect on the consequences for these particular groups and that they can trust the other group members in thoroughly applying these measures, too.

### Improving (Governmental) Initiatives and Measures

In Section “Norm Strategies for Optimizing Anti-Corona Measures,” the subjective quality and total normed values of the elements of our model were combined for the three examined anti-Corona measures to “automatically” deduce norm strategies according to the Means–End Theory of Complex Cognitive Structures. Across all of the measures to counter the COVID-19 pandemic and its consequences, a pattern emerged.

The protection of the close family and the wider family and friends from COVID-19 (attitude) as well as the practicability of anti-Corona measures in these social spheres (perceived behavioral control) are above average regarding both the relevance to people and the subjective quality. This accounts for all of the three measures (restrictions on outdoor activities, tips for hygiene, and tips for mental health) to counter the COVID-19 pandemic and its consequences. This means that the taken anti-Corona measures addressed the criteria that are most important for people to evaluate such measures relatively well (during the first phase of the pandemic). Therefore, from the people’s perspective, policymakers and related institutions can build on the recent measures in case of a similar crisis. They should analyze which elements of the recent initiatives led to a good protection of close social groups and made applying the measures in the daily lives feasible with relative ease. The identified elements of the recent measures should be used as the core of initiatives taken in case of a similar crisis in the future regarding both the measure itself and its communication and explanation to the people. At this point, however, it should be noted that the quality of the recent anti-Corona measures is evaluated relatively well by the people but not regarded as being perfect. A perfect evaluation would have meant values for the subjective quality of 100 on the scale 0 “not good” to 100 “very good.” Therefore, the recent anti-Corona measures still have room for improvement with regard to the protection and practicability within close social groups, even though it is relatively small compared to the other social spheres.

The people attribute an above-average relevance to protecting colleagues at work and the society in general from COVID-19 (attitude) but, with single exceptions across the three examined anti-Corona measures (protection of the society in general by the restrictions on outdoor activities and protection of colleagues at work by the tips for mental health), a below-average quality to the recent measures regarding these social spheres. This means that, based on the people’s views, the recent anti-Corona measures have to be assessed with the aim of finding ways to improve their effectiveness in protecting larger groups with relatively loose social ties. The practicability of the recent measures (perceived behavioral control) in the context of work and societal life in general is of above-average importance to the people. With a single exception across the three examined anti-Corona measures (practicability of the tips for hygiene within the social sphere of colleagues at work), the quality of these measures is rated below average by the people. Thus, the recent anti-Corona measures have a relative weakness with regard to people being able to easily integrate a corresponding behavior in their work and wider social life. This means in this area, too, that policymakers and related institutions should identify parts and elements of the recent initiatives that can increase the protection of larger social entities and are, at the same time, relatively easy to be implemented in the people’s daily lives. Because of the relatively high relevance perceived by the people, improving both the protection of colleagues at work and the society in general as well as the practicability of measures in these social spheres should be given a high priority for potential future crisis, similar to the current one.

The willingness to fulfill the expectations of others (subjective norm) is of substantially lower relevance to the people than the two afore-discussed aspects. Against this backdrop, policymakers and related institutions are advised to abstain from integrating any form of social pressure in initiatives like the recent anti-Corona measures [see also section “People’s Expectations on (Governmental) Initiatives and Measures”]. An option for future (governmental) reactions to a severe crisis might be to encourage the people to communicate with each other. This should be, however, considered with a lower priority. The focus should be on protecting people from a threat and making it as easy as possible for them to realize restrictive measures in their lives.

### Limitations and Outlook

Our data revealed that, in contrast to other studies that investigated healthy behavior like the use of bicycle helmets or seat belts (Lajunen and Räsänen, 2004; Şimşekoğlu and Lajunen, 2008), the subjective norm is of lower relevance to the people in the context of the COVID-19 pandemic and the referring counter-measures [see section “People’s Expectations on (Governmental) Initiatives and Measures”]. However, it has to be mentioned that a comparison of daily life healthy behavior can probably only partially be compared to an exceptional situation of a global pandemic. Nonetheless, we suggest that future research should focus more on the subjective norm when examining the Covid-19 pandemic and healthy behavior in general, having the aforementioned limitation in mind. One reason for the divergence of others and our findings might be that we collected our data in Germany and cannot rule out a cultural influence on the results. Therefore, we suggest that our study is replicated in other countries. Such a replication should not aim to find a one-fits-all solution on how to deal with severe crises, like the COVID-19 pandemic, all over the globe but to find solutions that are suited best for the specific expectations of people in different cultures.

Our findings indicate that the three examined anti-Corona measures and all of its subordinated elements are received rather well by the people [see section “People’s Evaluation on (Governmental) Initiatives and Measures”]. In this context, it has to be mentioned that we collected the data at an early stage of the Corona crisis in Germany after the measures to counter the pandemic were newly introduced. Therefore, we cannot make any statements about if and how the attitudes toward the anti-Corona measures have changed. Therefore, a longitudinal research approach based on our method is advised to reveal the people’s evaluation of the long-term effects of the severe measures, which deeply impact the lives of virtually everyone.

It is fair to assume that the results from our study give solid and reliable insights in the perception of the anti-Corona measures of the average German, as our participants are not only students but also fully integrated in the work life. However, our sample is rather homogenous regarding, among other indicators, age or circumstances of life. Therefore, we could not make any statements about how rather stable characteristics of people, like personality dispositions or the individual situation of life, influence the perceived relevance and quality of anti-Corona measures. Against this backdrop, it might be fruitful to examine this aspect in experimental designs using our model or elements of it.

We could find that the subjective relevance and the perceived quality of the protection from the COVID-19 pandemic and its consequences (attitude), the willingness to fulfill the expectations of others (subjective norm), and the practicability of anti-Corona measures (perceived behavioral control) are showing similar patterns [see section “People’s Evaluation on (Governmental) Initiatives and Measures”]. One explanation can be that the media reporting about the COVID-19 pandemic and the counter-measures has formed or at least influenced the expectations of people. We regard a deeper examination of this aspect as worthwhile, especially to gain a better understanding how the media influences people’s opinions in times of crisis.

In Sections “Norm Strategies for Optimizing Anti-Corona Measures” and “Improving (Governmental) Initiatives and Measures,” we pointed out on which evaluation criteria of the people a government or related institutions should focus when securing or improving the effectiveness of the recent measures to counter the COVID-19 pandemic. Our method did not allow us to specifically pinpoint single elements of the three anti-Corona measures to be persevered or modified. Against this backdrop, we suggest that, in future research, the evaluation criteria of the people are correlated with the elements of the main anti-Corona measures which can contribute to an improved design and communication of (governmental) initiatives countering potential severe crises in the future.

### Conclusion

One of the main results of our research is that the protection from the COVID-19 pandemic and its consequences (attitude) and the practicability of the anti-Corona measures (perceived behavioral control) are more important to the people than the willingness to fulfill the expectations of others (subjective norm), as discussed in Section “People’s Expectations on (Governmental) Initiatives and Measures.” This indicates that policymakers should focus on the utility to people when designing and communicating measures that severely impact people’s lives. Furthermore, a factual and comprehensive communication of the taken initiatives is advised. Even though all of the social spheres are relevant to the people in the context of the COVID-19 pandemic, the close family shows the highest importance from the people’s perspective. Thus, the close family should be at the core of (governmental) measures in times of crisis.

The perceived quality of the anti-Corona measures shows a similar pattern as subjective relevance, as discussed in Section “People’s Evaluation on (Governmental) Initiatives and Measures.” This indicates that the German government took measures that structurally mirror the expectations of the people. It, however, can also indicate that the media coverage and governmental statements influenced the expectations of the citizens. Furthermore, we could find that the restrictions on outdoor activities and tips for hygiene are evaluated better than the tips for mental health, which indicates that people focus on immediate threats rather than long-term consequences during a severe crisis.

In Section “Improving (Governmental) Initiatives and Measures,” we discussed options of improving the recent anti-Corona measures. In the case of a similar crisis like the current one, (governmental) initiatives can be built on the recent measures with regard to the close family and the wider family and friends, so that the effectiveness in these areas should be secured with a high priority. The effectiveness of anti-Corona measures with regard to protecting colleagues at work and the society in general and their practicability in these social spheres should be increased with a high priority. Social pressure or similar approaches, on the other hand, should not or only with low priority be included in initiatives during a crisis like the COVID-19 pandemic.

Apart from the people’s evaluation of anti-Corona measures, we could develop a three-level model that can potentially be used in future research of the COVID-19 pandemic, health-related behavior in the social context, and societal crises and counter-measures in general (see section “Hypothesized Model and Research Questions”). The same accounts for our method, the Means–End Theory of Complex Cognitive Structures (see section “Materials and Methods”), which allows to model and measure cognitions or attitudinal systems with multiple levels. The comparison of empirically measured and calculated values (see section “Subjective Calculated Quality of the Attitude, Subjective Norm and Perceived Behavioral Control, and Social Spheres”) and the comparison of our results in Section “Results” and the results of partial least-square path modeling (see Supplementary Materials) indicate a good adequacy of our model and method.

## Data Availability Statement

The raw data supporting the conclusions of this article will be made available by the authors, without undue reservation, to any qualified researcher.

## Ethics Statement

Ethical review and approval was not required for the study on human participants in accordance with the local legislation and institutional requirements. The patients/participants provided their written informed consent to participate in this study.

## Author Contributions

HG and SR-F designed and planned the study and wrote the manuscript. HG processed the data, performed the statistical analysis, and designed the figures and tables. LH conducted literature search and contributed to writing the manuscript, especially the introduction. All authors provided critical feedback and helped in every stage of the research, analysis, and manuscript.

## Conflict of Interest

The authors declare that the research was conducted in the absence of any commercial or financial relationships that could be construed as a potential conflict of interest.
