# Supplementary material for: How People Evaluate Anti-Corona Measures for Their Social Spheres: Attitude, Subjective Norm, and Perceived Behavioral Control
Source: Front Psychol. 2020 Nov 12;11:567405. doi: 10.3389/fpsyg.2020.567405 (PMC7689201; doi:10.3389/fpsyg.2020.567405)
Supplement: Supplementary file 1 [file Table_1.pdf]

Table S1: Results of the partial least path square modelling with the calculated quality as dependent variable and the attitude, subjective norm and perceived behavioral control as independent variables of the inner model: R squared, goodness of fit index (GoF, geometric mean of the average communality and the R squared value), path coefficients and p-values, and variance inflation factors (VIF) and weights of the variables of the outer model (n = 663) (own representation).

**Restrictions on outdoor activities (caluclated quality)**	**R squared**	**GoF**			
	0.928	0.752			
**Inner model**			**Outer model**		
**Construct**	**Path coefficient**	**p-value**	**Indicator**	**VIF**	**Weight**
Attitude	0.494	4.37E-186	Close family	2.256	0.294
			Wider family and friends	2.748	0.296
			Colleagues at work	2.197	0.279
			Society in general	2.444	0.297
Subjective norm	0.184	2.31E-66	Close family	1.984	0.378
			Wider family and friends	2.265	0.355
			Colleagues at work	1.483	0.303
			Society in general	1.289	0.229
Perceived beahavioural control	0.115	1.54E-153	Close family	1.159	0.369
			Wider family and friends	1.373	0.348
			Colleagues at work	1.206	0.361
			Society in general	1.419	0.364
**Tips for hygiene (caluclated quality)**	**R squared**	**GoF**			
	0.893	0.761			
**Inner model**			**Outer model**		
**Construct**	**Path coefficient**	**p-value**	**Indicator**	**VIF**	**Weight**
Attitude	0.514	6.86E-105	Close family	2.535	0.289
			Wider family and friends	3.000	0.295
			Colleagues at work	2.231	0.283
			Society in general	2.188	0.288
Subjective norm	0.228	2.12E-29	Close family	2.390	0.334
			Wider family and friends	2.620	0.320
			Colleagues at work	2.075	0.304
			Society in general	1.620	0.244
Perceived beahavioural control	0.375	1.38E-100	Close family	1.233	0.355
			Wider family and friends	1.431	0.368
			Colleagues at work	1.486	0.339
			Society in general	1.471	0.343
**Tips for mental health (caluclated quality)**	**R squared**	**GoF**			
	0.971	0.878			
**Inner model**			**Outer model**		
**Construct**	**Path coefficient**	**p-value**	**Indicator**	**VIF**	**Weight**
Attitude	0.469	1.35E-203	Close family	4.738	0.277
			Wider family and friends	5.816	0.282
			Colleagues at work	3.649	0.266
			Society in general	3.120	0.262
Subjective norm	0.249	3.79E-87	Close family	3.959	0.297
			Wider family and friends	4.904	0.302
			Colleagues at work	2.789	0.268
			Society in general	2.228	0.255
Perceived beahavioural control	0.410	3.16E-217	Close family	2.238	0.308
			Wider family and friends	2.957	0.299
			Colleagues at work	2.435	0.274
			Society in general	2.409	0.284
