# Supplementary material for: How People Evaluate Anti-Corona Measures for Their Social Spheres: Attitude, Subjective Norm, and Perceived Behavioral Control
Source: Front Psychol. 2020 Nov 12;11:567405. doi: 10.3389/fpsyg.2020.567405 (PMC7689201; doi:10.3389/fpsyg.2020.567405)
Supplement: Supplementary file 4 [file Table_4.pdf]

Table 4: Results of the partial least path square modelling with the empirical quality as dependent variable and the attitude, subjective norm and perceived behavioral control as independent variables of the inner model: R squared, goodness of fit index (GoF, geometric mean of the average communality and the R squared value), path coefficients and p-values, and variance inflation factors (VIF) and weights of the variables of the outer model (n = 663) (own representation).

**Restrictions on outdoor activities (empirical quality)**	**R squared**	**GoF**			
	0.456	0.526			
**Inner model**			**Outer model**		
**Construct**	**Path coefficient**	**p-value**	**Indicator**	**VIF**	**Weight**
Attitude	0.494	2.47E-35	Close family	2.256	0.339
			Wider family and friends	2.748	0.301
			Colleagues at work	2.197	0.234
			Society in general	2.444	0.291
Subjective norm	0.184	6.60E-07	Close family	1.984	0.427
			Wider family and friends	2.265	0.333
			Colleagues at work	1.483	0.250
			Society in general	1.289	0.253
Perceived beahavioural control	0.115	2.14E-04	Close family	1.159	0.459
			Wider family and friends	1.373	0.301
			Colleagues at work	1.206	0.294
			Society in general	1.419	0.385
**Tips for hygiene (empirical quality)**	**R squared**	**GoF**			
	0.487	0.562			
**Inner model**			**Outer model**		
**Construct**	**Path coefficient**	**p-value**	**Indicator**	**VIF**	**Weight**
Attitude	0.413	1.12E-20	Close family	2.535	0.279
			Wider family and friends	3.000	0.302
			Colleagues at work	2.231	0.278
			Society in general	2.188	0.295
Subjective norm	0.281	6.23E-11	Close family	2.390	0.343
			Wider family and friends	2.620	0.311
			Colleagues at work	2.075	0.298
			Society in general	1.620	0.250
Perceived beahavioural control	0.088	6.50E-03	Close family	1.233	0.306
			Wider family and friends	1.431	0.394
			Colleagues at work	1.486	0.301
			Society in general	1.471	0.393
**Tips for mental health (empirical quality)**	**R squared**	**GoF**			
	0.539	0.653			
**Inner model**			**Outer model**		
**Construct**	**Path coefficient**	**p-value**	**Indicator**	**VIF**	**Weight**
Attitude	0.462	3.49E-26	Close family	4.738	0.281
			Wider family and friends	5.816	0.282
			Colleagues at work	3.649	0.254
			Society in general	3.120	0.269
Subjective norm	0.198	5.24E-06	Close family	3.959	0.309
			Wider family and friends	4.904	0.306
			Colleagues at work	2.789	0.252
			Society in general	2.228	0.253
Perceived beahavioural control	0.157	5.41E-06	Close family	2.238	0.342
			Wider family and friends	2.957	0.286
			Colleagues at work	2.435	0.248
			Society in general	2.409	0.289
